# Bone marrow infiltrated Lnc-INSR induced suppressive immune microenvironment in pediatric acute lymphoblastic leukemia

**DOI:** 10.1038/s41419-018-1078-8

**Published:** 2018-10-11

**Authors:** Yaping Wang, Xiaoyun Yang, Xiaoyan Sun, Liucheng Rong, Meiyun Kang, Peng Wu, Xiaohui Ji, Rufeng Lin, Jie Huang, Yao Xue, Yongjun Fang

**Affiliations:** Department of Hematology and Oncology, Children’s Hospital of Nanjing Medical University, Nanjing Medical University, 72# Guangzhou Road, Nanjing, Jiangsu Province China

## Abstract

Immune escape due to immunosuppressive microenvironments, such as those associated with regulatory T (Treg) cells is highly associated with initial occurrence and development of solid tumors or hematologic malignancies. Here, we employed high-throughput transcriptome screening to demonstrate immunosuppression-associated increases in the long noncoding (lnc) RNA lnc-insulin receptor precursor (INSR), which was corrected with INSR expression in CD4+ T cells extracted from the bone marrow of patients with childhood acute T lymphoblastic leukemia. Loss-of-function and gain-of-function assays in vitro and in vivo revealed that membrane-localized and cytoplasm-localized lnc-INSR promoted Treg distribution and decreased the percentage of cytotoxic T lymphocytes, which induced tumor growth. Through direct binding with INSR, lnc-INSR blocked the INSR ubiquitination site, causing abnormal activation of INSR and the phosphatidylinositide 3-kinase/AKT-signaling pathway. These results indicated that lnc-INSR might promote immune suppression by enhancing Treg-cell differentiation and serve as valuable therapeutic targets in the immunosuppressive tumor microenvironment.

## Introduction

Acute lymphoblastic leukemia (ALL) is an aggressive hematologic malignancy arising from the hematopoietic precursors of the lymphocytes^1^. It is most common in childhood, with an annual incidence rate of 42 cases per 1 million children under age 15, with ~15% of ALL cases in children being T cell ALL (T-ALL)^2–4^. Despite the development of diagnostics or treatment approaches in clinical and experimental oncology, the prognosis for T-ALL remains unfavorable^5^.

Bone marrow (BM) represents the site of initiation, progression, and frequently recurrence of leukemia, and within the marrow space, tumor cells occupy the same niche that supports healthy hematopoiesis, allowing the capacity to respond to cues in that niche that regulate diverse processes, including hematopoietic cell quiescence^6,7^. This is consistent with current concepts regarding the critical role of the tumor microenvironment in the pathogenesis of hematologic malignancies^8,9^. B and T lymphocytes, plasma cells, dendritic cells, neutrophils, and macrophages reside in BM stroma and parenchyma, and the BM regulates immune cells through the production of cytokines, chemokines, and growth factors^10,11^. The cross-talk between immune cells and malignant cells or the cytokines secreted by either immune cells or malignant cells formed the immune microenvironment (IME)^12,13^. Immune escape and tolerance in the tumor microenvironment are closely involved in tumor progression, caused by T cell exhaustion, and mediated by inhibitory signals based on the activation of immune-checkpoint molecules, including programmed death-1 (PD-L1), cytotoxic T lymphocyte-associated protein 4, and T cell immunoglobulin and mucin domain-containing-3 (TIM-3)^14–16^.

For the IME in solid tumors, tumor-infiltrating lymphocytes (TILs) and peripheral blood lymphocytes (PBLs) are two major components^17^. Multiple lines of evidence show that TILs are manifestations of host immune reactions against cancers^18,19^. An increased population of regulatory T (Treg) cells was reported in TILs of patients with ovarian cancer, lung cancer, breast cancer, esophageal cancer, and liver cancer^20,21^. For the IME in leukemia, a significantly increased percentage of Treg cells was observed in the BM of B- and T-ALL patients, implicating it as a poor prognostic factor^22,23^. Although high-throughput transcriptomic and proteomic approaches are being employed to interrogate immune surveillance and escape mechanisms in patients with solid tumors and identify actionable targets for immunotherapy, our knowledge of the immunological landscape of hematological malignancies, as well as our understanding of the molecular circuits underlying the establishment of immune tolerance, is not comprehensive.

Long noncoding (lnc) RNA is transcribed from a large proportion of the human genome and plays a crucial role in the development of human carcinoma and congenital diseases by pre-transcriptional, transcriptional, or post-transcriptional regulation^24^. The function of lncRNAs in the immune system has also been well-documented, with lnc-epidermal growth factor receptor (EGFR) promoting the differentiation of Treg cells in the Hepatocellular carcinoma (HCC) immune microenvironment through an EGFR-independent approach^25^. However, the landscape of transcriptome alteration, including lncRNA and mRNA, in the IME of pediatric T-ALL patients remains unclear.

In this study, we conducted high-throughput screening, including mRNA and lncRNA, of the T cell-infiltrated BM of pediatric T-ALL patients and healthy volunteers, and examined the potential function and detailed mechanism of lncRNA in the immune microenvironment associated with leukemia development.

## Results

### Transcriptome landscape of BM T cells from T-ALL children and healthy volunteers

**Fig. 1:**
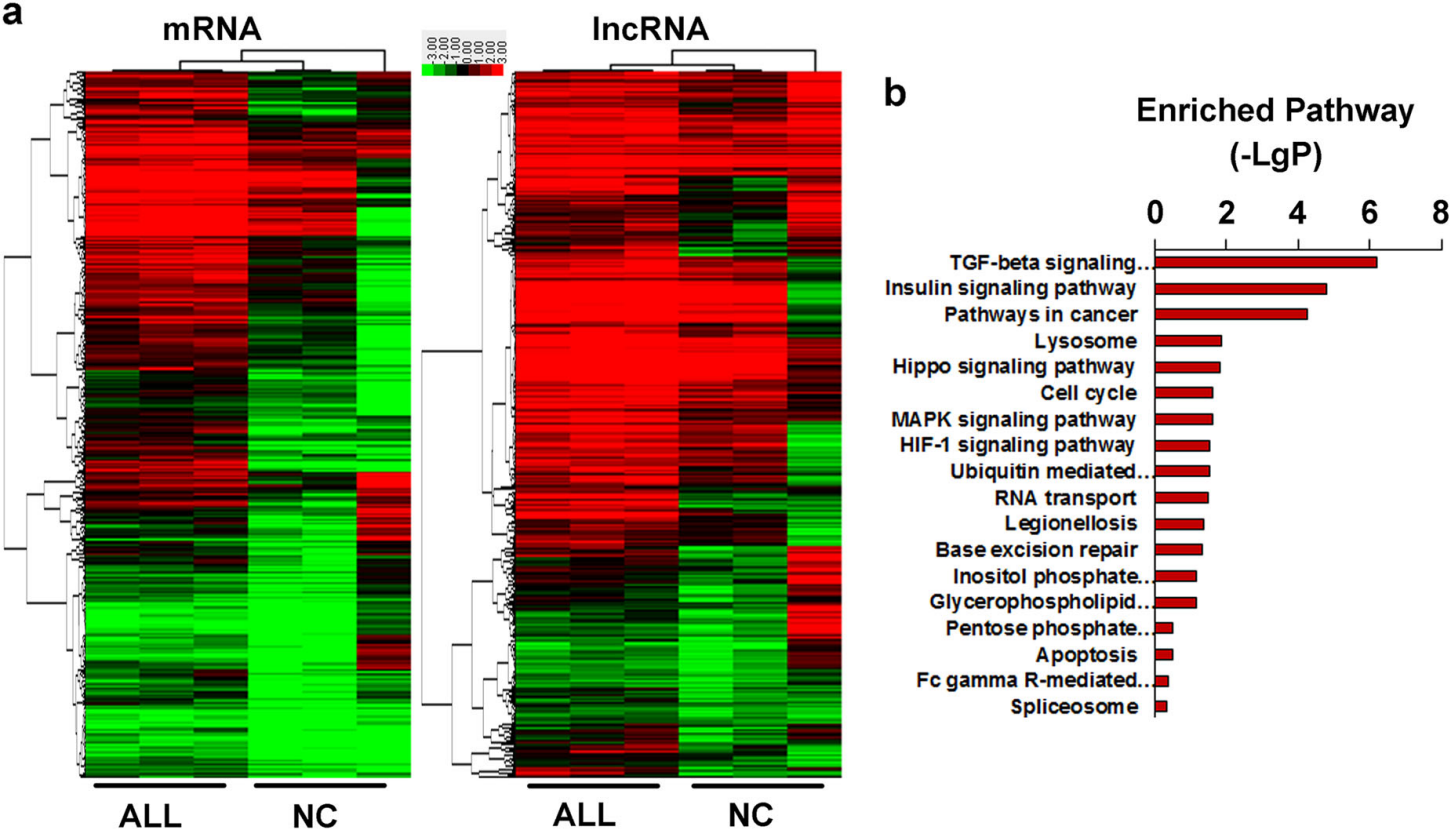
The expression profiling of transcriptome in T cells from pediatric T-ALL and healthy controls. **a** The different expressed lncRNA and mRNA was clustered by using hierarchical clustering analysis with the cut-off 4/0.25 and false discovery rate < 0.05 in T cells extracted bone marrow from paediatric T-ALL (labeled as ALL) and healthy controls(labeled as NC). CD3 beads was used for sorting the T cells after culturing for 3 day with IL-2 supplied. The green/red color bar indicated the relative expression level of mRNA/lncRNA presented by log-transformed. **b** Pathway enrichment was conducted by using different database. *P* < 0.05 was used as cutoff with log-transformed

BM from three patients diagnosed with T-ALL based on MICM and three healthy volunteers was collected, and T cells were sorted using anti-CD3 magnetic beads in mononuclear cells (MCs) extracted from six BM samples. The high-throughput microarray integrated with both mRNA and lncRNA was applied for screening differential expression profiles between T-ALL patients and controls. Aberrant expression of mRNA or lncRNA underwent hierarchical clustering using a heat map, resulting in a profile of the differential expression of mRNA and lncRNA in the T cells of T-ALL children (Fig. 1a). Among these, we identified 881 increased mRNAs, 277 decreased mRNAs, 204 increased lncRNAs, and 128 decreased lncRNAs according to a fold-change cut-off of 4/0.25. mRNAs exhibiting aberrant expression were employed for further pathway enrichment (Fig. 1b), which showed that among the 18 enriched signaling pathways, the transforming growth factor (TGF)-β-signaling pathway, insulin-signaling pathway, and pathways in cancer represented the three most significant (*p* < 0.0001) results for 100 differentially expressed mRNAs.

**Fig. 2:**
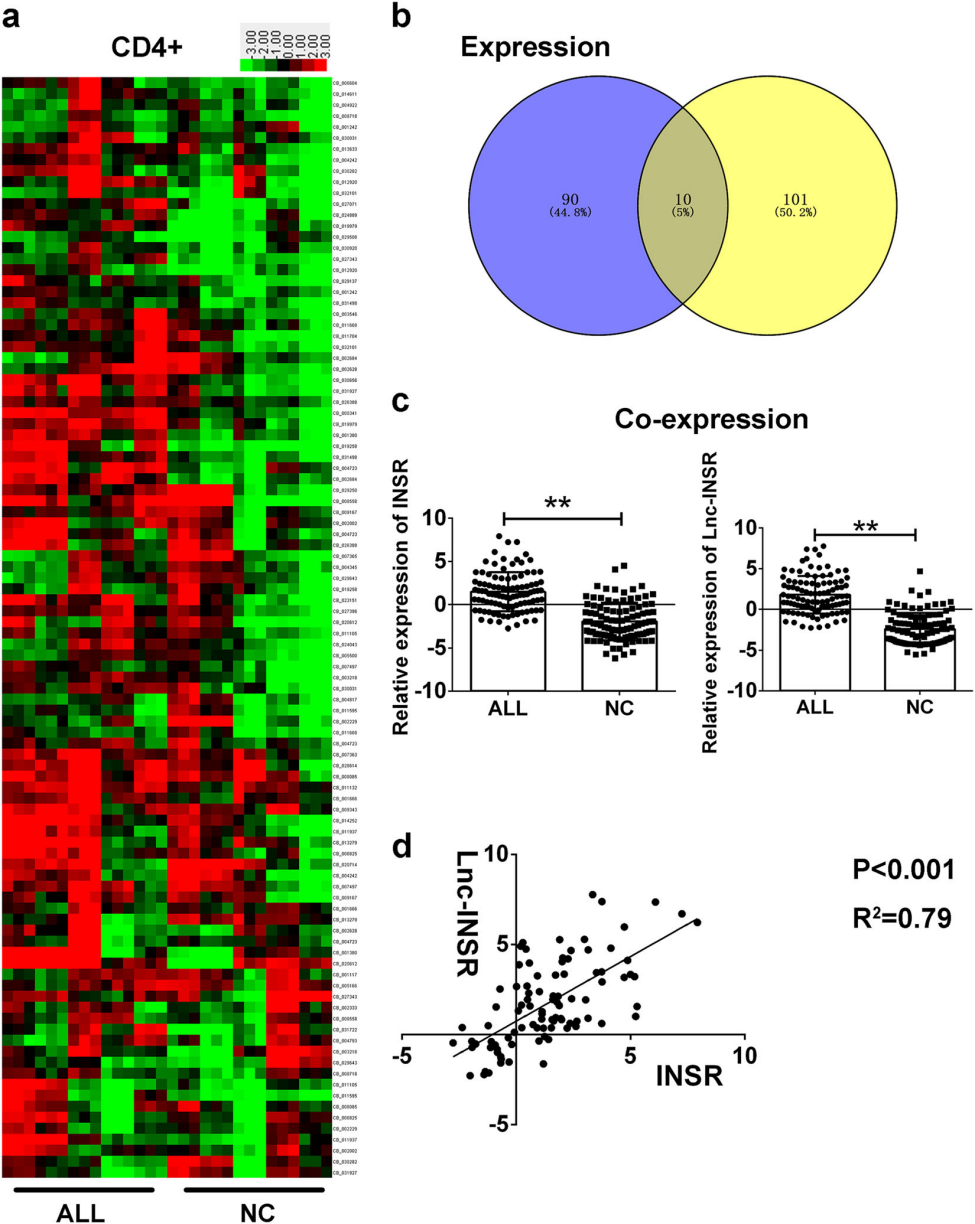
Lnc-INSR corrected expression with INSR in T helper cells **a** Cells were purified with CD3 and CD4 beads. The candidate mRNAs through step by setp screening was further detected by RT-PCR in 15 paired T helper cells extracted from bone marrow of T-ALL patients and healthy controls. The green/red color bar presented by heat map indicated the relative expression level of mRNA/lncRNA presented by log-transformed ranking from −3 to 3. **b** The candidate ten mRNA was screened from the venny of different expression (*p* < 0.05) and co-expression mRNA (*R* > 0.99). **c** Relatively expression level of lnc-INSR and INSR T helper cells of ALL children and health controls. Data was log-transformed as presented with mean ± SEM. **d** Pearson analysis was performed in calculating the correlation of lnc-INSR and INSR with log-transformed data. Data was presented with mean ± SEM (** indicated *p* < 0.01)

To focus on T helper cells, especially CD4+ T cells, we used the human anti-CD4 antibody to investigate the potential population harboring the aberrantly expressed mRNAs. Next, we started detecting the 100 candidate mRNA obtained from the pathway enrichment analysis in 15 paired ALL and control samples. CD4+ T cells were sorted using the CD3 and CD4 antibodies in MCs extracted from 15 BM samples from children diagnosed with T-ALL and healthy controls. The relative expression of mRNAs detected with RT-PCR are presented in a heat map (Fig. 2a), and co-expression correlation analysis was applied using the 1158 abnormally expressed mRNAs and 332 lncRNAs. A correlation value cut-off of 0.99 resulted in another 111 mRNAs used as input. After screening candidate mRNAs against the 100 mRNAs enriched in the pathways described in the previous section, 10 mRNAs were identified as being potential targets of lncRNAs and involved in critical pathways (Fig. 2b). We then compared the expression of the 10 candidate mRNAs in CD4+ T cells from 15 T-ALL and controls, finding that three mRNA including INSR, IL-6R, and MET showed significantly differential expression with the *p* < 0.05 (Supplementary Figure 1a and b), with the insulin receptor precursor (INSR) being most significant (*p* = 0.021) (Fig. 2c, left).

A larger sample size, including 100 CD4+ T cells, was extracted from BM MCs from T-ALL children and 100 healthy controls. INSR upregulation was confirmed, and its correlation with lncRNA, also known as TCONS_00011506, was investigated according to their increased levels in T-ALL immune cells (Fig. 2c, right). The Pearson’s correlation analysis was also conducted, resulting in strong correlations (*p* < 0.001; *R*^2^ = 0.79) between INSR and TCONS_00011506 (Fig. 2d). We subsequently referred to TCONS_00011506 as lnc-INSR based on their potential interaction. Since the lnc-INSR was obtained through the microarray screening. The detailed transcript names is annotated as TCONS_00011506 or ENST00000504928.1. It was located in chr 6:53429095–53448131 with the size of 482 bp. Besides, clinicopathological relevance analysis revealed the high correlation between the increased WBC count/ blasts BM percentage and higher expression of lnc-INSR and INSR (Supplementary Table 1)

### Membrane and cytoplasmic localization of lnc-INSR and co-localization with INSR and forkhead box P3 indicates a positive correlation with Treg distribution

**Fig. 3:**
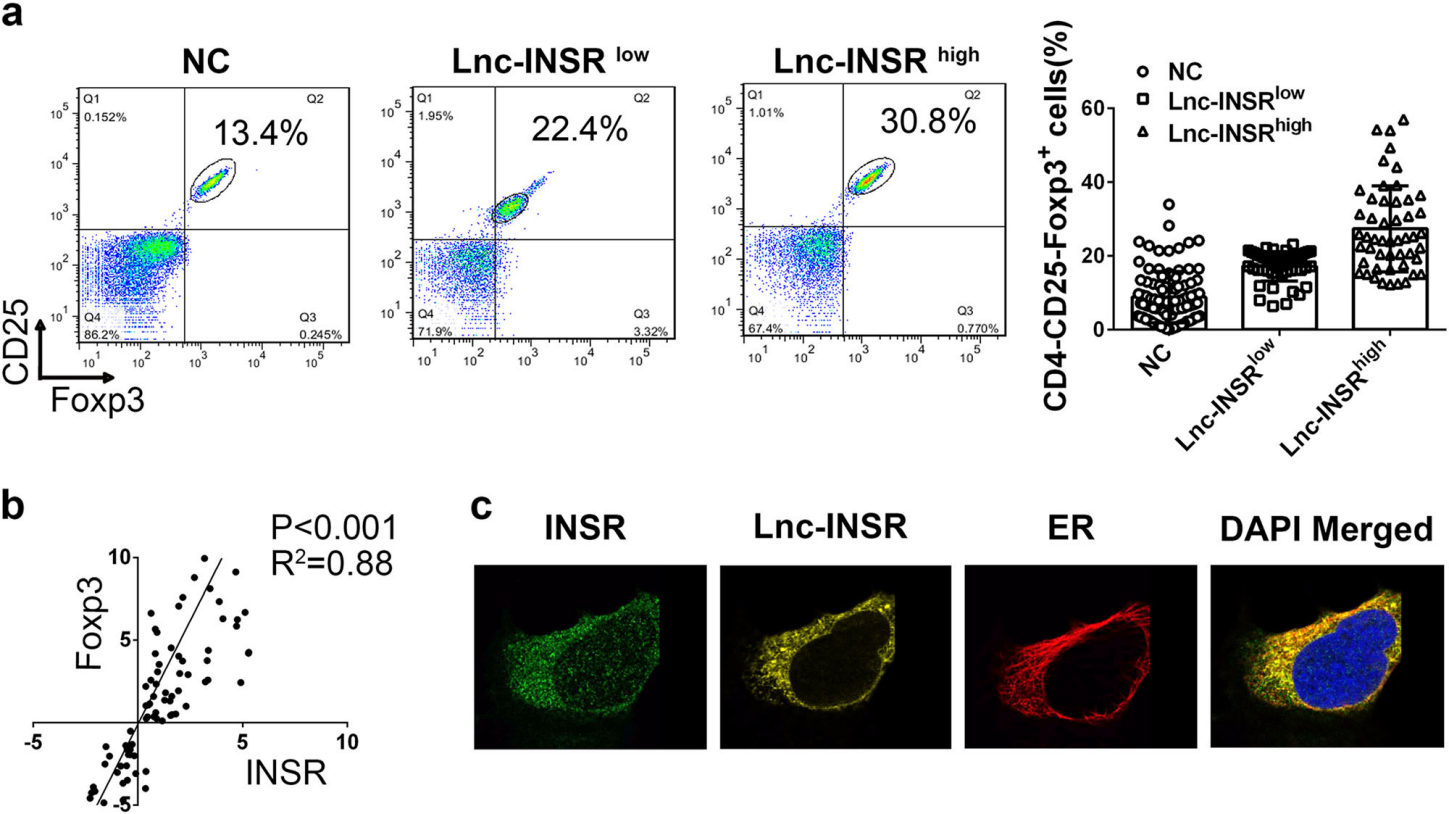
The membranal and cytoplasmic lnc-INSR associated with Treg distribution. **a** The percentage of Treg in the infiltrated CD4 (+) T cell was detected by flow cytometry in 96 paired clinical samples CD4, CD25, and Foxp3 was used as biomarker for Treg. **b** Pearson correlation analysis between the Treg distribution and the expression of lnc-INSR. **c** The subcellular location of lnc-INSR was examined by FISH in T cells extracted from T-ALL children. INSR location was detected by IF. ER stained with red was applied as membrane marker. DAPI was used for control stain. INSR was stained with green, lnc-INSR was yellow

Previous studies reported that Treg distribution in the IME of ALL patients was associated with poor prognosis. Immune tolerance is executed partly by Foxp3+ Treg cells, which suppress autoreactive T cells^26^. More importantly, the functional role of the INSR-signaling pathway in Treg distribution remains contradictory. In this study, based on the abnormal expression of INSR and lnc-INSR in CD4+ cells, we investigated the potential functions of INSR and lnc-INSR in Treg differentiation. First, CD3+ T cells were divided for RT-PCR detection and flow cytometry investigation. For the samples from the 100 patients and 100 controls described previously, we confirmed the distribution of Treg cells (CD4+CD25+Foxp3+). The percentage of Treg cells in 94 T-ALL BM samples (six were unavailable for flow cytometry investigation due to low volume) was higher as compared with controls, further dividing the T-ALL patient samples according to increases in lnc-INSR expression Treg-cell percentage (Fig. 3a). Additionally, a positive correlation between lnc-INSR and forkhead box P3 (Foxp3) was observed (Fig. 3b). Immunofluorescence was then used to investigate co-localization of lnc-INSR and INSR in CD3+CD4+ T cells. Results showed that the membrane-localized lnc-INSR signal converged with that for INSR (Fig. 3c), indicating possible interaction between lnc-INSR and INSR involved in Treg-cell differentiation, ER was applied as membrane marker.

### Lnc-INSR direct binding with the cytoplasmic domain of INSR

**Fig. 4:**
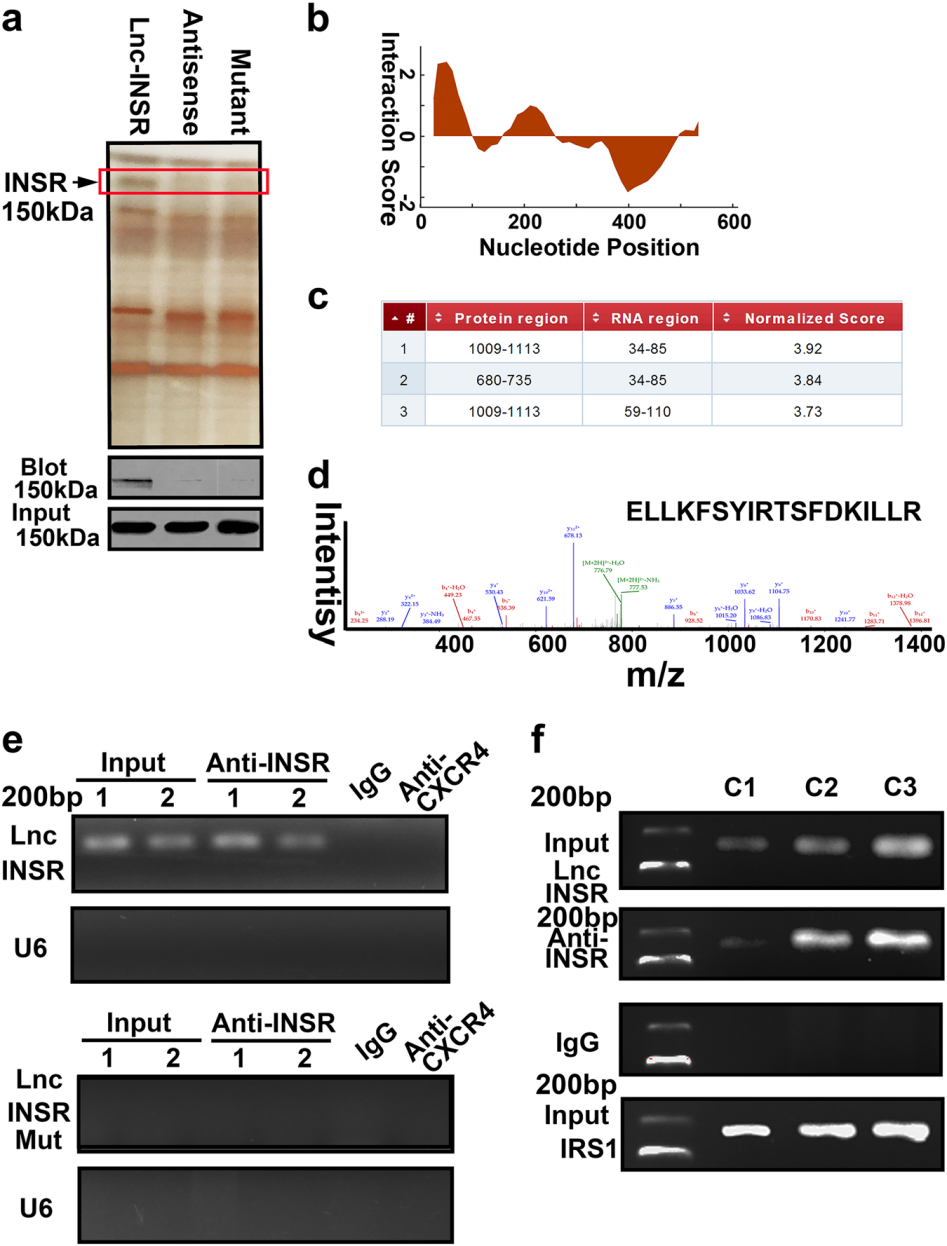
Lnc-INSR direct binding with INSR at 1009–1113aa **a** RNA pull-down experiment with CD4 (+) T cell cytoplasmic extract in different groups was presented as silver staining. Specific bands were identified by immunoblot of INSR. **b** The predicted binding site for lnc-INSR with INSR analyzed by *CatRAPID*. **c** Detailed binding site located in the RNA sequence of lnc-INSR and protein residue of INSR. **d** Mass spectrometry identification of special amino acid of INSR. **e** RIP assay was performed using INSR antibody and was validated by agarose electrophoresis by using different primer. CXCR4 antibody and U6 primer was used as negative control. **f** Cells were treated with different concentration of lnc-INSR lentivirus (C1 indicate 1U, C2 indicated 2U, and C3 indicated 3U comparing with the basal 20 TU/ml according to manufacturer). IRS1 was used as positive controls

We next performed a pull-down assay with biotinylated lnc-INSR to examine the potential binding protein for lnc-INSR. The antisense of lnc-INSR was used for control group. The silver staining presented a specific band at ~150 kDa for cells treated with biotinylated lnc-INSR (Fig. 4a). Based on this, bioinformatics software was applied by using *CatRAPID* to predict the detailed binding region. The main region in lnc-INSR were identified which could potentially interact with the INSR protein (Fig. 4b, c). We next constructed the mutant lnc-INSR at the predicted region, and further re-conducted the RNA pull-down assay, we found treating the cells with mutant type failed to capture the band at the ~150 kDa position. The detailed peptide fragment sequence captured by RNA pull-down was located in the cytoplasmic domain of INSR (1009–1113 amino acids) through blast analysis (Fig. 4a). Further mass spectrometric analysis and peptide fragment blasting confirmed the protein as INSR (Fig. 4d). Correspondingly, we also conducted the RNA immunoprecipitation (RIP) assay using an antibody targeting INSR. RIP further verified the specificity of this interaction **i**ndicating lnc-INSR, instead of the mutant lnc-INSR, may regulate INSR activity. The antibody targeting CXCR4 and IgG was used as the negative control (Fig. 4e). Next, CD4+ T cells were transduced with different doses of lnc-INSR lentiviral particles and the association of lnc-INSR with INSR was determined with RIP assay using anti-INSR antibodies and qPCR for lnc-INSR. The IRS1, also known as Insulin receptor substrate 1. It was identified as tyrosine phosphorylation of the insulin receptors or IGF-1 receptors, upon extracellular ligand binding, induces the cytoplasmic binding of IRS-1 to these receptors, through its PTB domains, thus, the IRS1 was used as positive control. Further dose response analysis indicated that the association of lnc-INSR with INSR was in a dose-dependent manner (Fig. 4f).

### Lnc-INSR enhances the activation INSR and blocks INSR ubiquitination

**Fig. 5:**
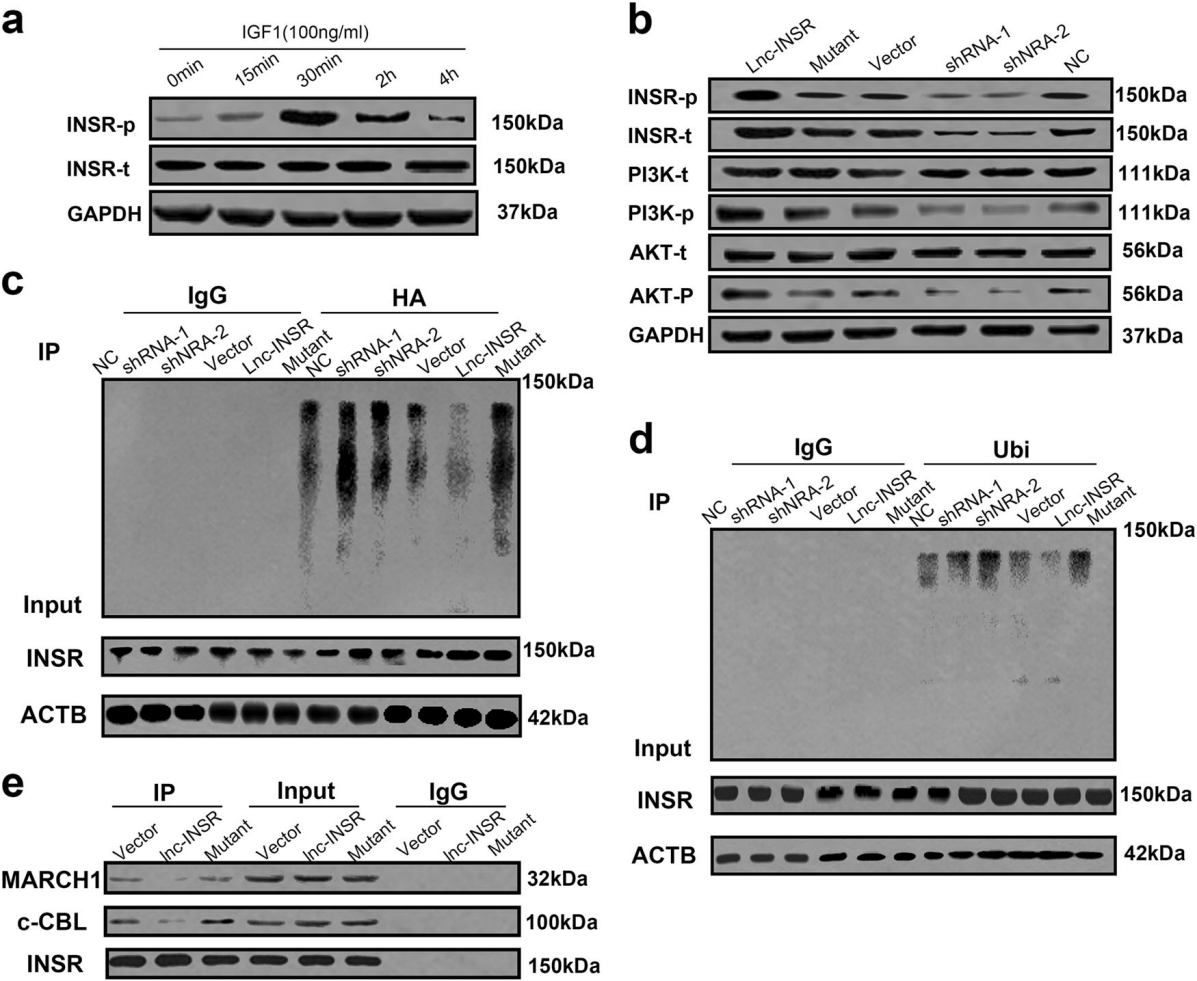
Lnc-INSR promoted the activation of INSR and PI3K/AKT-signaling pathway by blocking the ubiquitination induced degradation **a** Time dependent activation of INSR was investigated within conventional T cell at different time spots including 0 min, 15 min, 30 min, 2 h and 4 h after IGF1 (100 ng/ml) treatment by western blot. **b** INSR associated signaling alternation after different treatment with lnc-INSR overexpression or knock-down and was detected by western blot. **c** The immunoblot analysis of INSR ubiquitination. HA-ubiquitinated proteins were immunoprecipitated from T cells with lnc-INSR/INSR shRNA or overexpression lentivirus and HA-ubiquitin were immunoblotted for INSR and for HA-ubiquitin. Lysates were probed for the indicated proteins. **d** The immunoblot analysis of INSR ubiquitination. Ubiqitinated proteins immunoprecipitated from T cells with lnc-INSR/INSR shRNA or overexpression lentivirus were immunoblotted for INSR and for Ubiquitin. Lysates were probed for the indicated proteins. ACTB was used as controls (right panel). **e** The Co-IP assay was performed for investigate the binding of ubiquitin related protein (MARCH1 and c-CBL)

We then investigated whether INSR expression or downstream signal transduction involving INSR could be blocked or inactivated by lnc-INSR. Previous studies reported that INSR presents as dimer of two monomeric isomers (IR-A and IR-B)^27^. Insulin-like growth factor (IGF), as the main ligand for INSR associated with INSR function during in the pathogenesis of human malignant tumors, activates INSR (phosphorylation of Y1158) and the insulin receptor substrate, resulting in activation of phosphatidylinositide 3-kinase (PI3K)/AKT-signaling pathway^28–31^. Therefore, we detected levels of INSR phosphorylation under IGF1 stimulation. Because detection of INSR phosphorylation has not been investigated in conventional human T cells, we performed time-series analysis of phosphorylation activity at 0 min, 15 min, 30 min, 2 h, and 4 h post-IGF1 stimulation. We found that levels of phosphorylated INSR progressively increased within 30 min of stimulation, followed by a gradual deceased level. No difference was observed in total INSR expression during these periods (Fig. 5a).

**Fig. 6:**
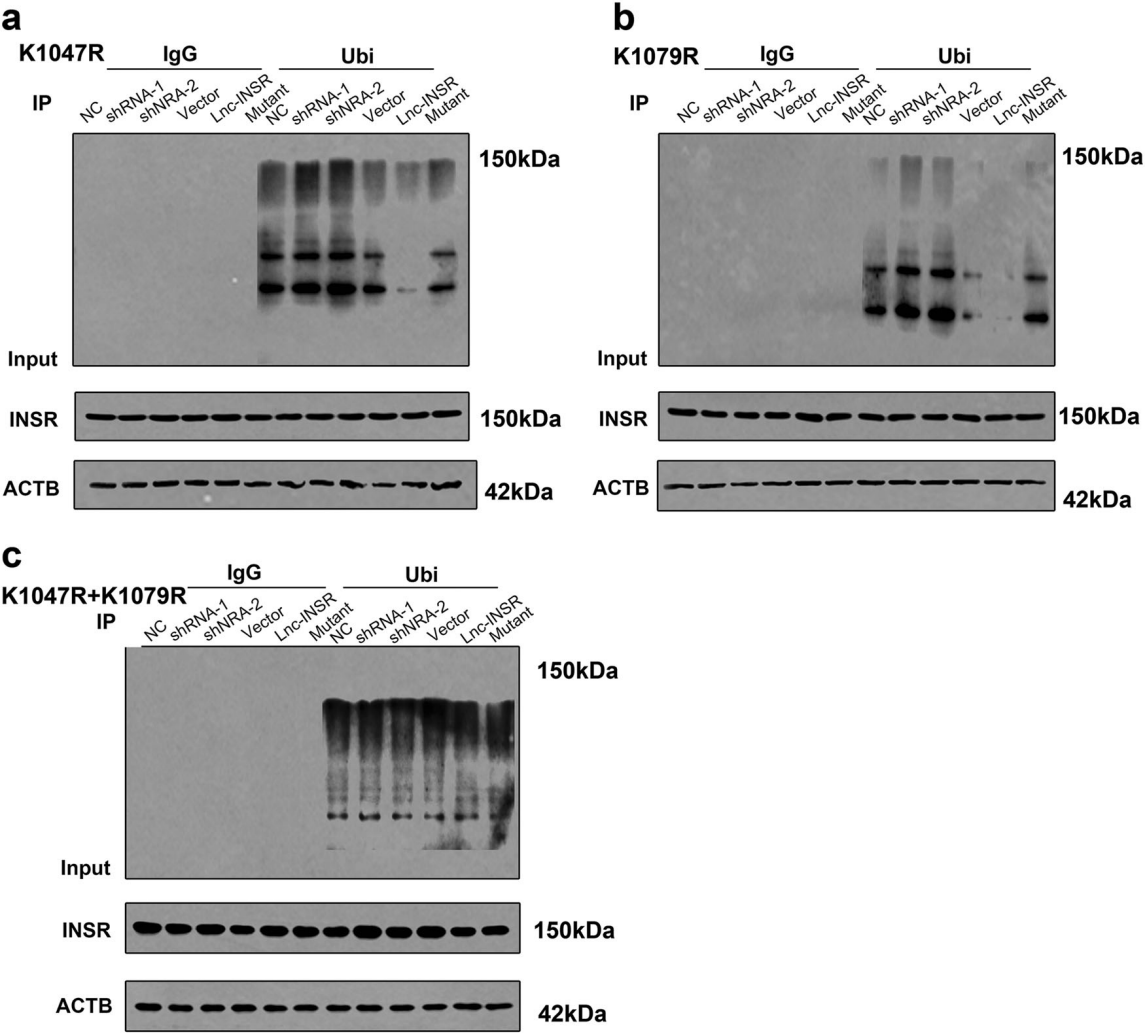
Mutant INSR K1047R and K1079R suppression of INSR induced by lnc-INSR. **a** Cells with mutant INSR K1047R were treated with lnc-INSR/INSR shRNA or overexpression lentivirus were immunoblotted for INSR and for ubiquitin. The ubiquitination could be suppressed by lnc-INSR. **b** Cells with mutant INSR K1079R were treated with lnc-INSR/INSR shRNA or overexpression lentivirus were immunoblotted for INSR and for ubiquitin. The ubiquitination could be suppressed by lnc-INSR. **c** Cells with mutant INSR K1047R and K1079 were treated with lnc-INSR/INSR shRNA or overexpression lentivirus were immunoblotted for INSR and for ubiquitin. No difference of the ubiquitination level was observed. Lysates were probed for the indicated proteins

We then performed loss-of-function and gain-of-function assays following IGF1 treatment within a 30-min window. Both overexpression and knockdown of lnc-INSR or INSR were confirmed with either RT-PCR or fluorescence microscopy (Supplementary Figures 1c-e and 2**)**. Compared with the control group, T cells infected with the wild-type lnc-INSR-overexpressing lentivirus significantly increased INSR phosphorylation at residue Y1158, whereas decreased lnc-INSR suppressed levels of both INSR and phosphorylated INSR. Moreover, the activation of INSR-mediated signaling was also detected upon T cell activation by treatment with anti-CD3 and CD28 beads based on observed increases in PI3K/AKT-signaling both the vector control and lnc-INSR-overexpressing groups (Fig. 5b). The detailed mutant sequence of lnc-INSR has been presented in Supplementary Figure 3. These findings suggested that lnc-INSR can bind to INSR protein to enhance activation of the PI3K/AKT pathway in conventional T cells. We then analyzed the functional domain of INSR to determine the mechanism of inactivation induced by lnc-INSR. Interestingly, we found ubiquitination sites (K1047 and K1079) in INSR, which agreed with previous studies reporting ubiquitination activity during signaling transduction and identifying the same ubiquitination sites involved in endocytic INSR degradation^28,32^. Based on this information, we hypothesized that this region might be affected by the binding region for lnc-INSR (amino acids 1009–1113). We then determined whether inactivated INSR and signal modulation by lnc-INSR expression were associated with INSR ubiquitination at K1047 or K1079. To rule out potential confounding effects of ectopic HA-ubiquitin expression, we measured endogenous polyubiquitination of INSR. We found lnc-INSR knockdown increased INSR ubiquitination, and that ectopic expression of wild-type, but not the mutant, lnc-INSR was associated with decreased INSR polyubiquitination in cells as presented in Fig. 5c. Consistent with our previous results, we also observed from the immunoprecipitation assay that lnc-INSR knockdown induced INSR polyubiquitination. Furthermore, we observed that lentiviral transfection with lnc-INSR and not mutant lnc-INSR suppressed INSR ubiquitination as presented in the right panel of Fig. 5d. To confirm interactions with ubiquitin ligases, pulldown assays using the INSR-specific antibody revealed interactions with membrane-associated ring-CH-type finger-1 (MARCH1) and a homologue of the casitas B-lineage lymphoma ligase (c-CBL). Furthermore, we observed decreases in MARCH1 and c-CBL levels in cells overexpressing lnc-INSR, whereas no difference in these levels was observed in cells transfected with the lnc-INSR mutant (Fig. 5e). To deeply test the role of lnc-INSR-mediated ubiquitination of INSR at Lys 1047 and Lys1079, we mutated Lys 1047 and Lys1079 to arginine (K1047R and K1079R). INSR K1047 and K1079R mutation presented no difference by upregulating, suppression or mutating lnc-INSR. We thought INSR K1047 and K1079R mutation may attenuate the ubiquitination suppression of INSR induced by lnc-INSR (Fig. 6a–c). These findings indicated that lnc-INSR binding to INSR may enhance activation of the PI3K/AKT pathway in conventional T cells by preventing polyubiquitination of residue K1047, K1079 in INSR, which inhibited INSR degradation. However, determination of the involvement of this process in the abnormal distribution of T cell subgroups in the T-ALL microenvironment requires further studied.

### Treg/CTL shift is induced by lnc-INSR dependent of INSR in vitro

**Fig. 7:**
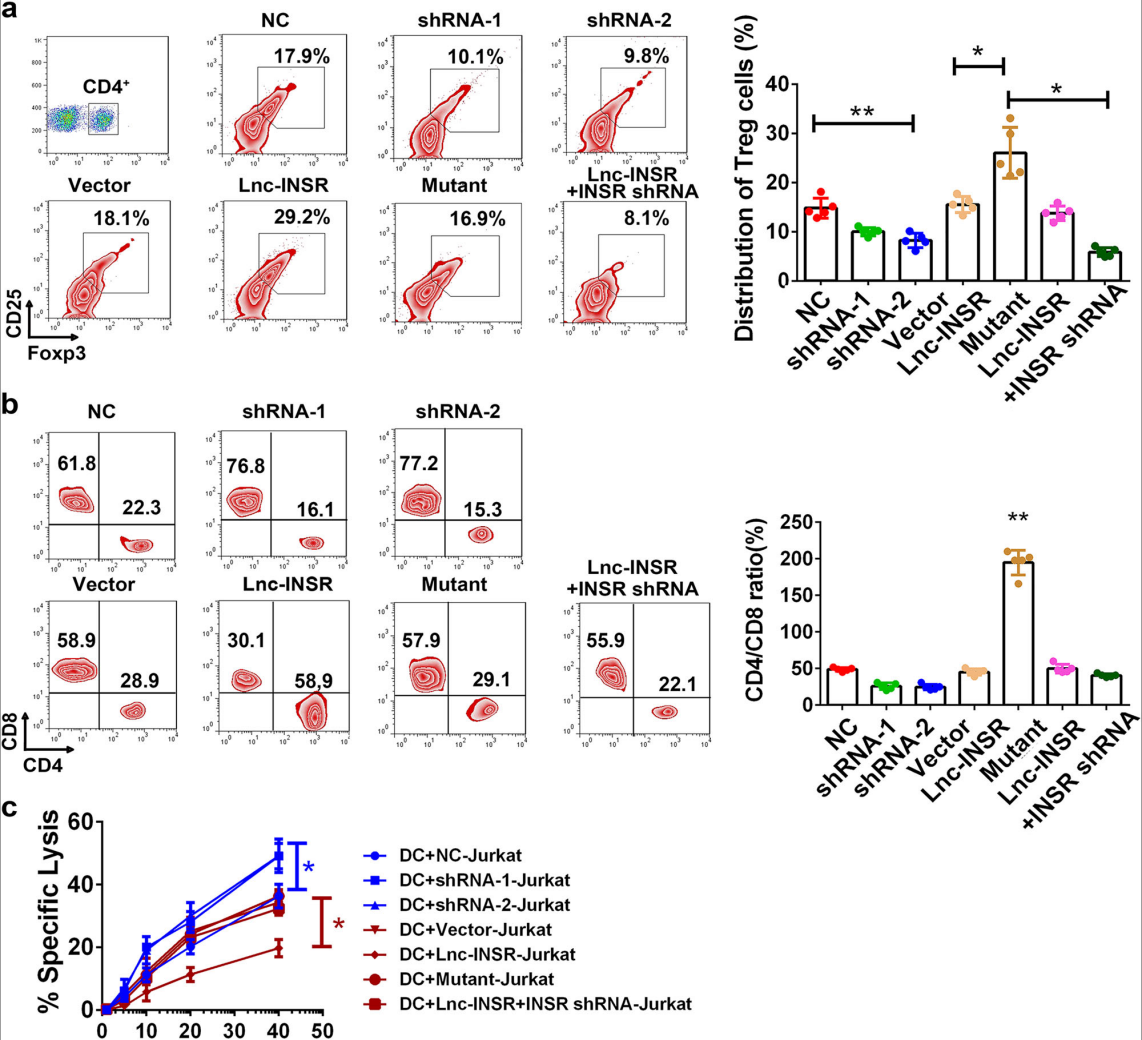
Lnc-INSR induced the Treg distribution while suppression the CTL function. **a** Different percentage of Treg in T cells treated with lnc-INSR in polarization stimulation assay. Quantitative calculation was listed in the right. **b** The CTL-suppression assay of Lnc-INSR was performed by mix culture of various types of CD4+ cell, CD8+, and OVA-induced DC cells. The ratio of cd4 and Cd8 cell ratio was evaluated by flow cytometry and presented in the right panel. **c** The cytolytic activity of the generated CTLs co-cultured with CD4 (+) cell treated with different groups was detected by ^51^Cr-release assay and was presented in the right panel with mean ± SEM (*n* = 5 each group, * indicated *p* < 0.05, ** indicated *p* < 0.01)

Because Treg cells are an immunosuppressive subgroup in the microenvironment of T-ALL children, we investigated alterations in Treg-cell distribution by modifying lnc-INSR expression. Using a polarization-stimulation assay in the presence of IGF1, cells were treated with TGF-β, anti-CD3, and CD28 beads for 7 days. We observed that the percentage of Treg cells decreased following suppression of lnc-INSR expression following treatment with two independent short-hairpin (sh) RNAs, but increased significantly upon lnc-INSR overexpression. Moreover, Treg-cell proliferation was attenuated by INSR knockdown (Fig. 7a).

In addition to altered Treg-cell distribution, we observed alterations in CD8+ cells in multiple IMEs of solid tumors. We then performed CTL-suppression assays involving ovalbumin (OVA)-induced dendritic cells co-cultured with equal proportions of mixed CD4+ and CD8+ cells sorted by beads. CD4+ and CD8+ cells were measured after co-culturing for 3 days, revealing decreases in the CD4+/CD8+ cell ratio following knockdown of lnc-INSR, whereas this ratio increased following lnc-INSR overexpression. Additionally, this ratio could be rescued by suppressing INSR expression (Fig. 7b). Furthermore, we performed a ^51^Cr-release assay to investigate the suppressive effect of lnc-INSR on CD4+ T cells. Jurkat cells were labeled with ^51^Cr, followed by co-culture with Jurkat-vaccinated dendritic cells or various CD4+ T cells. Decreased absorbance was observed in the group co-cultured with transfected CD4+ T cells, indicating stronger immunosuppressive activity by lnc-INSR-overexpressing cells. Additionally, knockdown of INSR expression also rescued immune suppressive activity, suggesting that lnc-INSR promoted an immunosuppressive microenvironment based on the presence of INSR (Fig. 7c).

### Lnc-INSR promotes tumor progression by promoting an immunosuppressive microenvironment in vivo

**Fig. 8:**
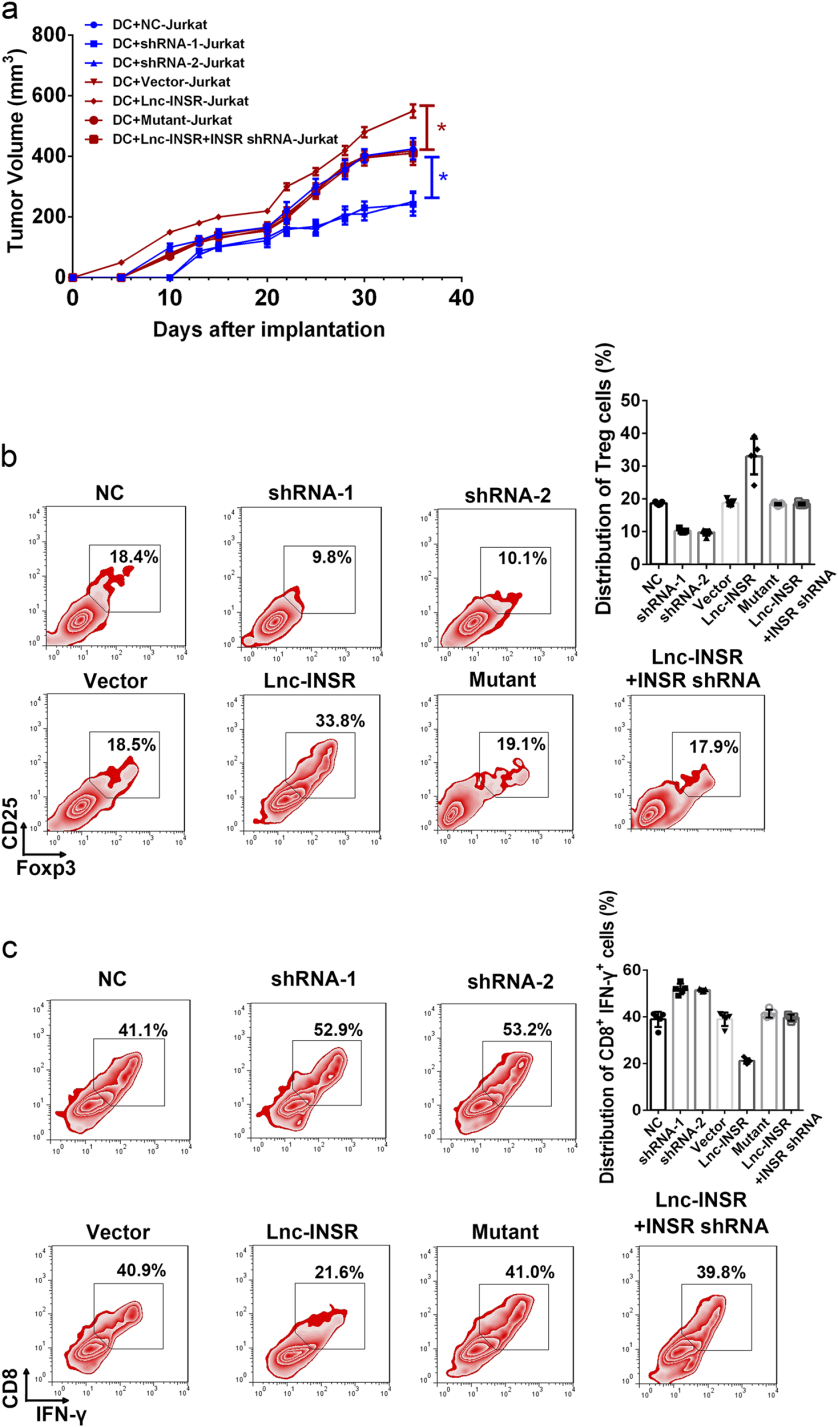
Lnc-INSR induced the tumor suppressive microenvironment promoting tumor growth in vivo. **a** Mice with established tumors were imaged every 7 d in different groups. Quantification of tumor growth was measured almost every 3 days. **b**, **c** The distribution of Treg and CTL was detected by FCM in T cells extracted from tumor in vivo model. Data was presented in the right panel with mean ± SEM

Mice homozygous for severe combined immune-deficiency (SCID) mutations and harboring a non-obese diabetic background (NOD/SCID) have been used in the majority of leukemia xenograft studies^33^. In the present study, NOD/SCID mice were used through subcutaneous injection of mixed cells, including modulated CD4+ T cell, CD8+ T cells, Jurkat cells, and dendritic cells vaccinated with Jurkat cells. Tumor growth was dramatically suppressed in mice administered CD4+ cells where lnc-INSR had been knocked down (Fig. 8a**)**. However, the antitumor effect was strongly attenuated in mice administered lnc-INSR-overexpressing CD4+ T cells, but could be rescued by the presence of mutant lnc-INSR or decreased INSR expression **(**Fig. 8a). Representative tumor burden in mice is also shown in Supplementary Figure 4. Further infiltrative immune cells in xenograft tumors were examined by flow cytometry following extraction. Consistent with in vitro results, the Treg distribution increased along with more aggressive tumor growth induced by lnc-INSR-overexpressing T cells accompanied by decreases in CTLs identified by CD8 and interferon (IFN)-γ staining. This phenotype could be reversed by infecting cells with a lentiviral vector containing an lnc-INSR mutant or INSR shRNA (Fig. 8b, c).

## Discussion

In solid tumors, the IME consists mainly of TILs, which are a type of white blood cell found in tumors^34,35^. TILs were previously thought capable of eliminating tumor cells^36,37^. Understanding the role of the immune system in the control of cancer and the mechanisms mediating immune evasion remain among the most challenging questions in tumor immunology. Immune cells are also important components of hematological malignancies. Immune responses are defective in patients with leukemia because of the presence of powerful immune-suppressive circuits activated by soluble factors and immune-checkpoint molecules, including PD-L1, TIM-3, and Indoleamine-pyrrole 2,3-dioxygenase-1^38,39^. Among immune cells, Treg cells dampen the functions of anti-neoplastic immune cells, thereby promoting cancer progression^40^. In this study, we mainly focused on lncRNA. Screening revealed the existence of lnc-INSR, and loss-of-function and gain-of-function assays confirmed that lnc-INSR could induce a suppressive IME by maintaining INSR stability and the associated activation of the PI3K/AKT-signaling pathway. Further investigation showed that this regulation was based on lnc-INSR inhibiting INSR ubiquitination. PI3K is preferentially enriched in leukocytes, and defects in its associated signaling pathway impairs T cell activation. Inhibition of PI3K/AKT-signaling blocks Treg-cell generation^41^. Additionally, during T cell regulation, T cell receptor signaling controls Foxp3 expression via PI3K/AKT^42^, indicating that this signaling network regulates de novo expression of Foxp3 in CD4+ T cells^43^.

To date, the important role of Treg cells in pediatric cancer has been mainly investigated in the context of graft-vs.-host disease;^44^ however, few in situ studies of Treg cells regarding the origins or development of pediatric T-ALL have been conducted, and little evidence identified regarding lncRNA function in the IME of pediatric T-ALL. Evidence exists showing that lncRNAs are involved in innate immune responses and T cell development, differentiation, and activation. For hematological malignancies, dysfunction of WT1-MEG3 signaling promotes leukemogenesis via p53-dependent and p53-independent pathways^45^, whereas Fas-antisense 1 lncRNA are negative regulatory factors for Fas^46^. INSR has been explored in human T cells. In INSR-knockdown LEW rats, Fischer et al. reported INSR as dispensable for Treg activity^47^, which inconsistent with our findings. This might be explained by their methods involving Treg-cell detection in the rat model instead of human myeloplasts, and using an INSR knockout model instead of a conditional INSR knockout. Additionally, the rat model was treated with 735 mg/kg doxycycline hyclate, which differed from our protocol. Furthermore, their model did not undergo hematological disease development. By contrast, Treg-cell induction in human mesenchymal stem cell culture supernatant is enhanced by the addition of IGF and suppressed by inhibition of IGF1 receptor^48^. Additional research is required in relation to the involvement of the human immune system and its direct role in pediatric leukemia. In this study, the ubiquitination site in INSR was predicted according to the binding site region of lnc-INSR. The K1047 and K1079 ubiquitination site was confirmed by other research. Tawo et al. found K1047 was preferentially used for INSR ubiquitination in vitro^28^ and Nagarajan et al. reported the MARCH1 ubiquitination of INSRβ Lys1079 controls INSRβ membrane stability^32^. Based on this, we thought the binding of lnc-INSR on INSR (amino acids 1009–1113) might associated with the polyubiquitination of INSR. We detected the ubiquitination status of INSR in cells treated with lnc-INSR overexpression or knock-down.

In conclusion, we reported discovery of a novel lncRNA, lnc-INSR, which blocks the ubiquitination site of INSR, thereby causing sustained activation of INSR and the PI3K/AKT-signaling pathway and resulting in promotion of an IME in the BM accompanied by shifts of Treg cells to CTLs. This environment was capable of inducing more aggressive tumor growth in leukemic cells. Our results suggested that candidate lncRNAs might represent efficacious therapeutic targets against IMEs associated with pediatric T-ALL.

## Materials and methods

### Clinical samples

Bone marrow samples were obtained from new-diagnosed with pediatric T-ALL patients receiving therapy at Children’s Hospital of Nanjing Medical University (Nanjing, China) during 2009 to 2017. Individuals with concurrent autoimmune disease, HIV, or syphilis, patients who received the immunosuppressive therapy for at least 1 month or patients diagnosed with immunodeficiency disease were excluded. Clinical characteristics were classified and diagnosed according to the guidelines of the Morphologic, Immunologic, Cytogenetic, and Molecular biologic classification technique (MICM). The healthy control samples were obtained from healthy volunteers or the children received the bone marrow biopsy test whom were diagnosed without hematological system diseases. All research was performed in compliance with government policies and the Helsinki Declaration. Experiments were undertaken with the understanding and written consent by guardian. The investigators were blinded to the group allocation during the experiment. The microarray data has been uploaded to ArrayExpress (https://www.ebi.ac.uk/arrayexpress) with the accession codes: E-MTAB-6335.

### In situ hybridization

In situ hybridization (ISH) was performed by employing the ISH kit from Boster (Wuhan, China) as previously described. Cells in the clinical specimens (10 μm) were fixed and permeablized using xylenes, ethanol and protease to allow biotin-labeled probes to access. Slides were treated with 30% H2O2 and ddH2O with the ratio of 1: 10 for 5 min, and then the 3% citric acid diluted pepsase was applied to expose the fragment of nucleic acid for 20 s.The second fixation was followed by using 1% paraformaldehyde/0.1 M PBS. Next, the slides were incubated with pre-hybridization solution at 40 °C for 2 h and then with lncRNA target probes at 30 °C overnight followed by 2 washes with 2× saline sodium citrate (SSC). After blocking, biotin-labeled anti-digoxin was added and incubated for 60 min. Finally, slides were stained with DAB, dehydrated with 100% ethanol and xylene, and mounted in a xylene-based mounting media. The slides were recorded by Pannoramic SCAN (3D HISTECH, Budapest, Hungary) and analyzed by Pannoramic Viewer (3D HISTECH, Budapest, Hungary).

### Flow cytometry

T cells extracted from bone marrow from T-ALL children or healthy controls were stained with fluorochrome-conjugated Abs and then analyzed by flow cytometry. In brief, the T cell were stimulated with leukocyte activation cocktail (BD Pharmingen, CA, USA) at 37 °C for 5 h. The different antibody of T cell markers were added for incubation and then finally stained with intracellular markers. Data were acquired on BD FACSVerse™ flow cytometer (BD Pharmingen, CA, USA). The detailed antibodies information were described in Supplemental Table 4.

### RNA pull-down and mass spectrometry

The biotin-labeled lncRNA with wild type full length, mutant full length as well as the antisense was used as previous report by using Thermo Scientific Pierce RNA 3′ Desthiobiotinylation Kit (Thermo Scientific Pierce, CA, USA). The RNA pull-down was conducted by using The Magnetic RNA-Protein Pull-Down Kit (Thermo Scientific Pierce, CA, USA) according to the manufacturer’s instructions. In brief, labeled RNA was applied to bind to streptavidin magnetic beads and Incubate for 15–30 min at room temperature with agitation. The protein binding to RNA was conducted by using RNA–protein binding reaction mix buffer, then the RNA and protein complex was washed. The complex was resolved by SDS-PAGE, and specific bands were excised and analyzed by mass spectrometry. Proteins in bands were eluted and digested. Digests were analyzed by Orbitrap Velos Pro LC/MS system (Thermo Scientific, CA, USA). Data was analyzed by Proteome Discoverer and the resulting peak lists were used for searching the NCBI protein database with the Mascot search engine.

### RNA immunoprecipitation, co-immunoprecipitation, and site-directed mutagenesis

The RIP assay was performed by using EZ-Magna RIP™ RNA-Binding Protein Immunoprecipitation Kit (Millipore, MA, USA). In brief, after preparing the appropriate amount of complete RIP lysis buffer for the quantity of cells being harvested, RIP Lysis Buffer, protease inhibitor cocktail, and RNase inhibitor were added and kept it on ice. The magnetic beads was prepared for immunoprecipitation. For purified antibodies, 5 μg were used for per IP. The pulled RNAs were detected by reverse transcription PCR and quantitative PCR. The primer sequences are listed in Supplementary Table 2. Anti-CXCR4 was used for negative control. U6 RNA was used for negative control. Total RNAs (input controls) and IgG were assayed simultaneously to demonstrate that the detected signals were the result of RNAs specifically binding to INSR. The hIR K1048 and K1079R-GFP was generated through site-directed mutagenesis of hIR-GFP using specific primers (Supplementary Table 2).

The co-immunoprecipitation assay was applied by using Dynabeads® Co-Immunoprecipitation Kit (Thermo Scientific Pierce, CA, USA). First the antibody was applied for coupling to the Dynabeads^®^ Protein A/G. For co-immunoprecipitation, the cell sample was resuspended in an extraction buffer. The then the coupling antibody was added into lysis of cells. The pulled protein was detected by using WB. The The detailed antibodies information was described in Supplemental Table 4.

### Standard ^51^Cr-release assays (CTL assays)

The Jurkat cells were transfected with RNA and labeled with ^51^Cr sodium chromate in X-VIVO 20 medium for 1 h at 37 °C. Target cells (1 × 10^4^) were transferred to a well of a round-bottomed 96-well plate. Varying numbers of CTLs were added to a final volume of 200 μl and incubated for 4 h. At the end of the assay, supernatants (50.0 μl per well) were harvested. The percentage of specific lysis was calculated by formula as (experimental release − spontaneous release)/(maximal release − spontaneous release) × 100%. Spontaneous and maximal releases were determined in the presence of either X-VIVO 20 medium or 2% Triton X-100, respectively.

### In vivo model

Jurkat cells (1 × 10^6^ cells) in 100 μl of buffered saline were subcutaneously injected into the dorsal tissue of 5-week-old to 6-week-old male NOD/SCID mice. The Jurkat cells were authenticated by STR profile and were tested to avoid mycoplasma contamination. T cells (2 × 10^6^) was overexpressed with lnc-INSR wild type and the mutant type, and was polarized by tumor monocytes (2 × 10^6^) in 100 μl of buffered saline were subsequently injected into the inguen of the male mice on day 3 after the Jurkat inoculation. The mock vector was regarded as control group. Ten mice were treated in each group. Tumors were measured every week after implantation, and the volume of each tumor was calculated (length × width^2^ × 0.5). All mice were sacrificed 5 weeks afterwards for further analysis. The investigators were blinded to the group allocation during the experiment.

### Statistical analysis

Data were presented as mean ± SEM with no special instructions. *χ*^2^ tests and the Student’s *t*-test analysis of variance was used to evaluate statistical differences in demographic and clinical characteristics. Pearson correlation analysis was used to analyze the relationship of associated factors. Statistical analysis was performed using STATA 10.0 and presented with the GraphPad prism software (CA, USA). In all cases, *p* < 0.05 was considered significant.

## Electronic supplementary material

Supplementary Information
