# Supplementary material for: Bone marrow infiltrated Lnc-INSR induced suppressive immune microenvironment in pediatric acute lymphoblastic leukemia
Source: Cell Death Dis. 2018 Oct 11;9(10):1043. doi: 10.1038/s41419-018-1078-8 (PMC6181910; doi:10.1038/s41419-018-1078-8)
Supplement: Supplementary file 1 — Supplementary Information [file 41419_2018_1078_MOESM1_ESM.docx]

**SUPPLEMENTARY INFORMATION FOR:**

**Bone marrow infiltrated Lnc-INSR induced suppressive immune microenvironment in pediatric acute lymphoblastic leukemia**

Yaping Wang^1, #,^ *, Xiaoyun Yang ^1, #^, Xiaoyan Sun ^1, #^, Liucheng Rong^1^, Meiyun Kang^1^, Peng Wu^1, #^, Xiaohui Ji^1^, Rufeng Lin^1^, Jie Huang^1^, Yao Xue^1^, Yongjun Fang^1,^*

1. Department of Hematology and Oncology, Children's Hospital of Nanjing Medical University, Nanjing Medical University, 72# Guangzhou Road, Nanjing, Jiangsu Province, China.

^#^ These authors contributed equally to this work.

*Corresponding authors:

Dr. Yaping Wang, Department of Hematology and Oncology, Children's Hospital of Nanjing Medical University, Nanjing Medical University, 72 Guangzhou Road, Nanjing, Jiangsu Province, China.

Phone: 86-25-51554586, Fax: +86-25-83304239

E-mail: [wyp_0919@163.com](mailto:wyp_0919@163.com)

Dr. Yongjun Fang, Department of Hematology and Oncology, Children's Hospital of Nanjing Medical University, Nanjing Medical University, 72 Guangzhou Road, Nanjing, Jiangsu Province, China.

Phone: 86-25-51554586, Fax: +86-25-83304239

E-mail: [dryjfang@gmail.com](mailto:dryjfang@gmail.com)

**CONTENTS:**

**1.** **SUPPLEMENTARY MATERIALS AND METHODS**

**2. SUPPLEMENTARY FIGURES AND FIGURE LEGENDS**

**3. SUPPLEMENTARY TABLES**

**SUPPLEMENTARY MATERIALS AND METHODS**

**Isolation of lymphocyte from bone marrow**

BM were treated with Ficoll (BD Pharmingen, CA, USA) density gradient centrifugation. Dissociated cells were filtered through a 150-mm mesh and separated by Ficoll centrifugation, and the mononuclear cells were washed and resuspended in RPMI 1640 supplemented with 10% fetal bovine serum (FBS) (Gibco, CA, USA). Anti-CD3 or CD4 or CD8 magnetic Dynalbeads (Invitrogen Life Technologies, CA, USA) were used to purify different type T cells for further investigation.

**RNA extraction and quantitative reverse transcription PCR (RT-PCR).**

Total RNA was isolated with Trizol and purified with RNeasy Mini Kit (Qiagen, Hilden, Germany) according to the manufacturer’s protocol. The expression level of lncRNA or mRNA levels were detected by quantitative reverse transcription PCR using ABI 7900 ( Life Technologies, CA, USA). Primer pairs used were listed in **Supplementary Table 2**. The detailed shRNA sequence was presented in **Supplementary Table 3**. All the primers targeting the 100 candidate mRNAs in the screening phase were obtained from the PrimerBank database (https://pga.mgh.harvard.edu/primerbank/).

**Microarray detection and bioinformatics analysis**

For microarray detection, 1×10^6^ CD3^+^T cell sorted from each sample was applied. Total RNA from each sample was quantified by the NanoDrop ND-1000. For The lncRNA/mRNA crosslinked microarray was employed (Capitalbio, Beijing, China). The sample preparation and microarray hybridization were performed based on the manufacturer’s standard protocols with minor modifications. The 4/0.25 was used as cutoff after normalization with the control point. Agilent Feature Extraction software (version 11.0.1.1) was used to analyze acquired array images. Quantile normalization and subsequent data processing were performed using the GeneSpring GX v12.0 software package (Agilent Technologies, CA, USA). After quartile normalization of the raw data, lncRNAs and mRNAs, which had flags in Present or Marginal (“All Targets Value”) in at least 6 out of 9 samples, were chosen for further analysis. LncRNA and mRNA expression patterns were revealed via Hierarchical Clustering. Pathway analysis was performed via KEGG, Biocarta and Reatome software. The Fisher’s exact test and χ2 test were used to identify the significant pathways. The threshold of significance was defined by P-value and False Discovery Rate (FDR). The mRNA expression in the candidate pathway was validated by RT-PCR and lncRNA/mRNA co-expression network was built to identify the correlated lncRNAs, which were subsequently validated by RT-PCR.

For the co-expression network, the system was built according to the normalized signal intensity of expression of specific genes and lncRNAs. For each pair of mRNA-lncRNA or mRNA-mRNA, Pearson Correlation was employed and the significant correlation pairs were used to construct the network. When networks were sampled the degree centrality became the simplest and most important measure of a gene or lncRNA centrality within a network. Moreover, the Network Structure Analysis was carried out to locate core regulatory factors, which connected most adjacent mRNAs and lncRNA, and were determined by the degree differences between two class samples.

The detailed binding site for lnc-INSR in the protein amino acid (aa) sequence was conducted by using the full length of lncRNA and INSR aa sequence as input. The *CatRAPID* database was applied.

**Mutagenesis construction and lentiviral packaging**

The full-length and mutants of lnc-INSR were synthesized by Genscript Co. Ltd. (Nanjing, China). Then the sequences were sub-cloned into PLV-HA or PLV-Luc plasmid, and further packaged for lentivirus particles. Both the mutation and wild type sequences were inserted into the lentivirus vector –pLV (Clontech, CA, USA) to generate expression vectors. The detailed sequence information wan presented in **Supplementary Figure 3**. These expression vectors were mixed with lentivirus packaging Δ8.91 and envelope expressing VSV-G plasmids to generate lentivirus particles in 293T cells. Viral particles were concentrated by ultracentrifugation and expression vector titers were determined. The CD4+ T cells isolated from ALL patients were cultured with TAKARA GT-T551 medium supplied with human IL-2, and then transduced with lentivirus with desired expression vectors.

**DC generation**

For generation of ALL specific DCs, the peripheral blood mononuclear cells (PBMCs) were isolated by centrifugation over Ficoll. Enrichment of monocytes was performed by negative selection by using immunomagnetic beads. GM-CSF (50ng/ml; R&D Systems, Minneapolis, MN, USA) and IL-4 (50ng/ml; R&D Systems) was added to generate DCs. After 5 days of culture, cells were immunized with cell whole lysate of Jurkat cells for 48h, leading to fully mature DCs.

**Immunofluorescence and Fluorescence in situ hybridization (FISH)**

For immunofluorescence analysis, The T cells were extracted from T-ALL children and were stained with rabbit anti-human INSR, followed by staining with Alexa Fluor 488-conjugated anti-mouse IgG (1:500, Ab150117). For FISH analysis, cells were treated with xylenes, ethanol and protease respectively before probe access. Then, cells was incubated in pretreatment buffer for 15 min and rinsed in water. Next, target probes are hybridized to the lncRNA at 40°C for 2 hours, this pair of probes creating a binding site of a preamplifier. After this, the preamplifier is hybridized to the target probes at 30°C and amplified with 6 cycles of hybridization followed by 2 washes. Cells are counter-stained to visualize signal. DAPI was applied for nuclear staining. Positive cells were quantified using Image-Pro Plus software (Media Cybernetics, MD, USA) and detected by confocal microscopy (Zeiss, Oberkochen, Germany).

**SUPPLEMENTARY FIGURE LEGENDS**

**

**

**Supplementary Figure 1. Relative expression of candidate mRNAs and lnc-INSR treated with two independent shRNAs.**

(**a**): Relatively expression level of IL-6R in T helper cells of ALL children and health controls. Data was log-transformed as presented with mean ± SEM. (**b**): Relatively expression level of MET in T helper cells of ALL children and health controls. Data was log-transformed as presented with mean ± SEM. (**c**): Relative expression of lnc-INSR in cells treated with lnc-INSR overexpression lentivirus. (**d**): Relative expression of lnc-INSR treated with two independent shRNAs. (**e**): RNASE4 was used as control. Data was presented with mean ± SEM.


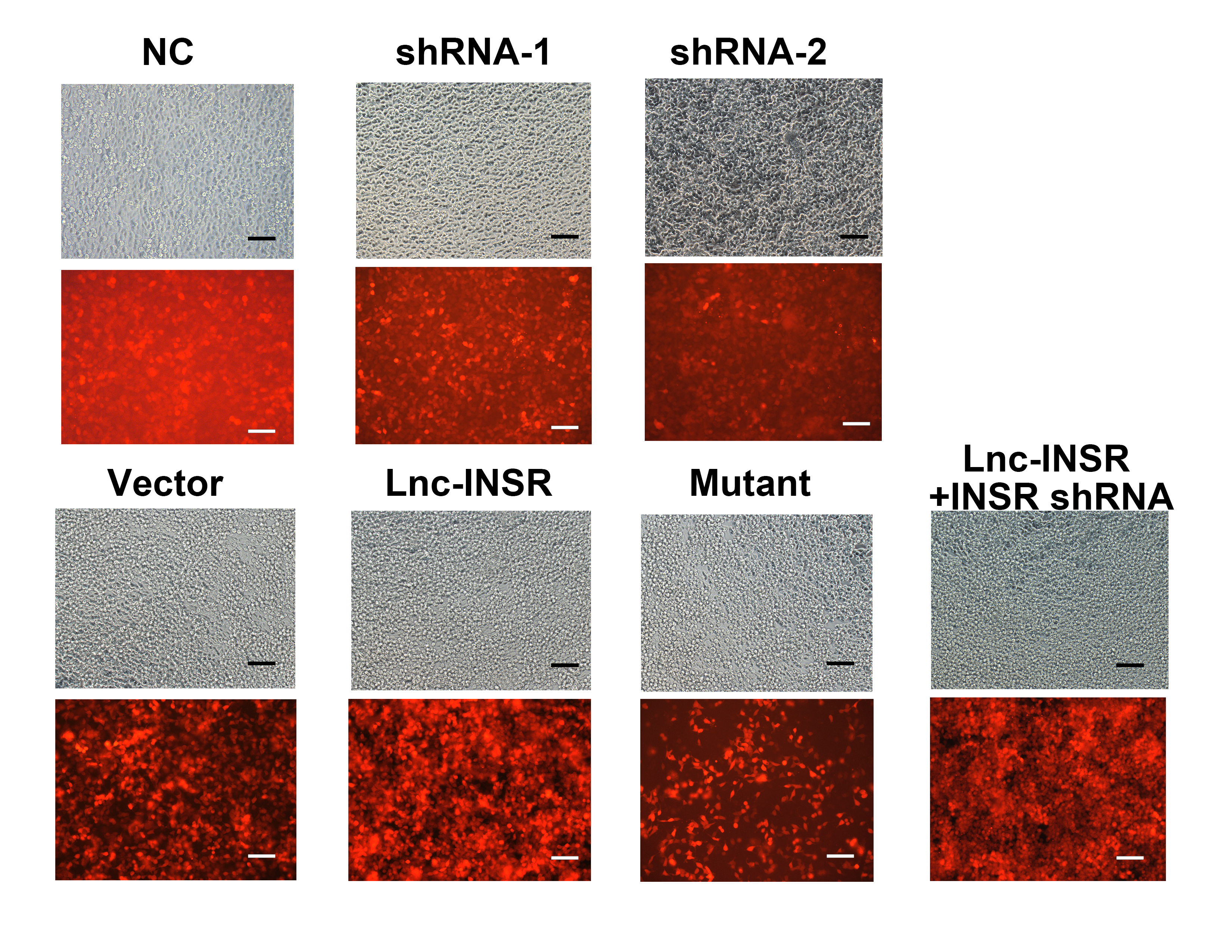


**Supplementary Figure 2. The efficiency detection of cell treated with different lentivirus.**

**
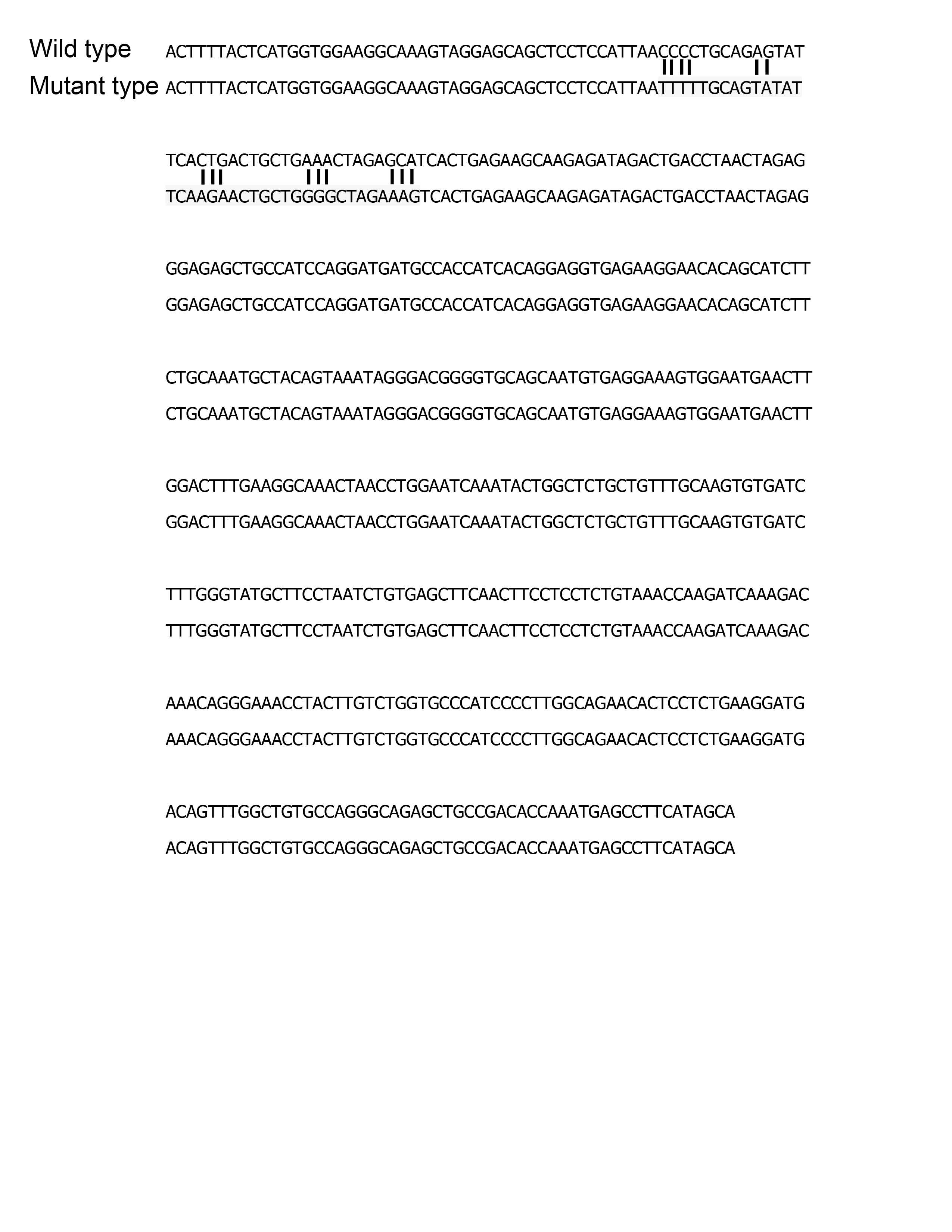
**

**Supplementary Figure 3. Detailed sequence information for the wild sequence and mutant sequence of Lnc-INSR.**

**
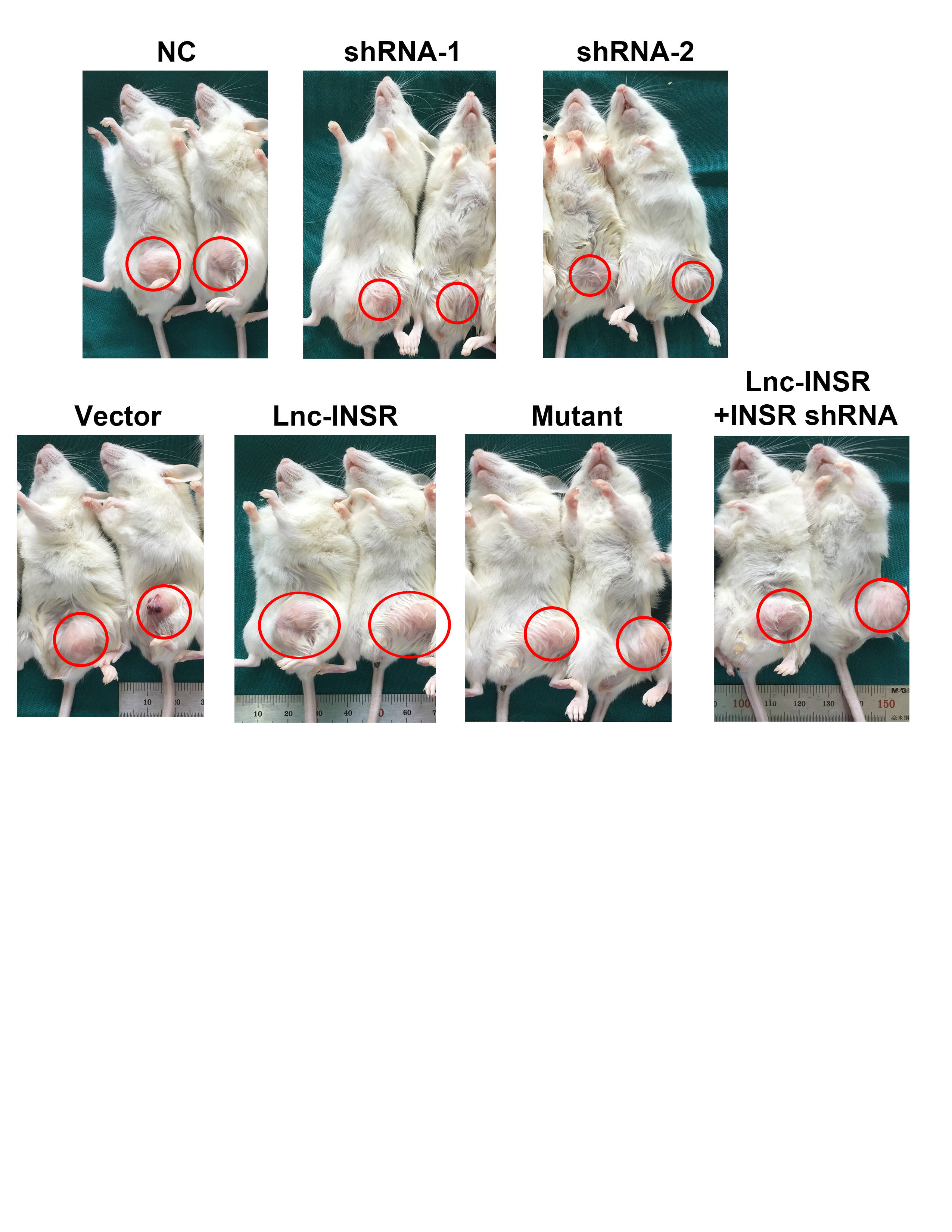
**

**Supplementary Figure 4. The representative tumor burden in mice after injected with mixed cells.**

The representative tumor burden in mice after injected with mixed cells. The red circle indicated the tumor position.

**SUPPLEMENTARY TABLES**

**Table1. The clinicopathological relevance analysis of Lnc-INSR and INSR expression in T-ALL children.**

|  | **Lnc-INSR** | | | | | **INSR** | | | | | | | |
| --- | --- | --- | --- | --- | --- | --- | --- | --- | --- | --- | --- | --- | --- |
| **Feather** | **Low** | **High** | **P value** | | | **Low** | | | **High** | | | **P value** | |
| **All cases** | 40 | 40 |  | | | 40 | | | 40 | | |  | |
| **Age(year)*** | 5.1(3.72) | 3.9(2.12) | 0.34 | 5.9(2.33) | | | 4.1(1.94) | | | 0.51 | | |  |
| **Gender** |  |  | 0.65 |  | | |  | | | 0.82 | | |  |
| Male | 24 | 22 |  | 23 | | | 22 | | |  | | |  |
| Female | 16 | 18 |  | 17 | | | 18 | | |  | | |  |
| **Mediastinal mass (%)** |  |  | 0.78 |  | | |  | | | 0.42 | | |  |
| Absent | 20 | 21 |  | 19 | | | 17 | | |  | | |  |
| Present | 11 | 10 |  | 11 | | | 15 | | |  | | |  |
| N/A | 9 | 9 |  | 10 | | | 8 | | |  | | |  |
| **Lymphadenopathy (%)** |  |  | 0.60 |  | | |  | | | 0.81 | | |  |
| Absent | 19 | 19 |  | 18 | | | 19 | | |  | | |  |
| Present | 9 | 12 |  | 10 | | | 12 | | |  | | |  |
| N/A | 12 | 9 |  | 12 | | | 9 | | |  | | |  |
| **Splenomegaly (%)** |  |  | 0.93 |  | | |  | | | 0.83 | | |  |
| Absent | 21 | 22 |  | 21 | | | 20 | | |  | | |  |
| Present | 12 | 12 |  | 14 | | | 12 | | |  | | |  |
| N/A | 7 | 6 |  | 5 | | | 8 | | |  | | |  |
| **Hepatomegaly (%)** |  |  | 0.38 |  | | |  | | | 0.61 | | |  |
| Absent | 15 | 14 |  | 18 | | | 17 | | |  | | |  |
| Present | 18 | 19 |  | 14 | | | 17 | | |  | | |  |
| N/A | 7 | 7 |  | 8 | | | 6 | | |  | | |  |
| **CNS involvement (%)** |  |  | 0.92 |  | | |  | | | 0.92 | | |  |
| Absent | 20 | 21 |  | | | 22 | | | 21 | | |  | |
| Present | 16 | 16 |  | | | 15 | | | 15 | | |  | |
| N/A | 4 | 3 |  | | | 3 | | | 4 | | |  | |
| **WBC count × 10^9^/L*** | 20.1(15.81) | 65.8(21.12) | **0.001** | | 22.1(19.2) | | | 77.3(29.22) | | | **0.002** | |  |
| **Blasts BM (%)*** | 65.2(12.85) | 91.8(6.91) | **0.007** | | 63.8(19.11) | | | 89.3(9.88) | | | **0.008** | |  |

CNS, central nervous system; WBC, white blood cell; PB, peripheral blood; BM: bone marrow. Missing values were excluded in the calculation of P-values. *indicated data presented with Mean ± SD and analyzed with student t test. *P* value in bold indicated statistically significant. The median expression level was used as the cutoff.

**Supplementary Table 2. Primers for Quantitative RT- PCR**

| **Gene name** | **All Patients** | **Sequence** |
| --- | --- | --- |
|  |  |  |
| Lnc-INSR | Forward Primer | CATCCCCTTGGCAGAACACT |
|  | Reverse Primer | GAAGGCTCATTTGGTGTCGG |
| INSR | Forward Primer | AAAACGAGGCCCGAAGATTTC |
|  | Reverse Primer | GAGCCCATAGACCCGGAAG |
| Foxp3 | Forward Primer | GTGGCCCGGATGTGAGAAG |
|  | Reverse Primer | GGAGCCCTTGTCGGATGATG |
| GAPDH | Forward Primer | GGAGCGAGATCCCTCCAAAAT |
|  | Reverse Primer | GGCTGTTGTCATACTTCTCATGG |
| RNASE4 | Forward Primer | GCTGTCGACCAGTGTCAAG |
|  | Reverse Primer | GCAGCCCAGGCAAAGAA |
| INSR K1047R | Forward Primer | GCAATGCCAGGGACATCATCAGGGGTGAGGCAGAGACC |
|  | Reverse Primer | GGTCTCTGCCTCACCCCTGATGATGTCCCTGGCATTGCCC |
| INSR K1079R | Forward Primer | AATGAGGCCTCGGTCATGAGGGGCTTCACCTGCCATCACGTG |
|  | Reverse Primer | CACGTGATGGCAGGTGAAGCCCCTCATGACCGAGGCCTCATT |

**Supplementary Table 3. Summary of shRNA Oligos**

| **Name** | **Oligo Sequence** |
| --- | --- |
| [INSR](http://www.abcam.cn/cd97-antibody-c-terminal-ab155798.html) shRNA 1 | CACCGGGCCTCCGCTCAGTATTTGTCGAAACAAATACTGAGCGGAGGCCC |
| [INSR](http://www.abcam.cn/cd97-antibody-c-terminal-ab155798.html) shRNA 2 | CACCGGTCGCGGGCGTGGAAGAGAACGAATTCTCTTCCACGCCCGCGACC |
| [INSR](http://www.abcam.cn/cd97-antibody-c-terminal-ab155798.html) shRNA 3 | CACCGCACGTGTGTGTGTCCATTAGCGAACTAATGGACACACACACGTGC |
| Lnc-INSR shRNA 1 | CACCGCAGGTGCCCGCCCGCCAGTCCGAAGACTGGCGGGCGGGCACCTGC |
| Lnc-INSR shRNA 2 | CACCGCCCGCAGATCGCGACCCAGACGAATCTGGGTCGCGATCTGCGGGC |
| Lnc-INSR shRNA 3 | CACCGCCACCACCGCAAGGGCCTCCCGAAGGAGGCCCTTGCGGTGGTGGC |

**Supplementary Table 4. Summary of antibodies**

| **Name** | **Catalog#** | **Company** |
| --- | --- | --- |
| [Anti-INSR antibody](http://www.abcam.cn/cd97-antibody-c-terminal-ab155798.html) | Ab983 | Abcam |
| Anti-INSR(Y1158) | Ab1158 | Abcam |
| Anti-PI3 Kinase P25 beta antibody | Ab180967 | Abcam |
| Anti-PI3 Kinase P25 beta (Y464) antibody | Ab138364 | Abcam |
| [Anti-AKT antibody](http://www.abcam.cn/grk2-antibody-ab47999.html) | [Ab182729](http://www.abcam.cn/grk2-antibody-ab153712.html) | Abcam |
| [Anti-AKT](http://www.abcam.cn/grk6-antibody-ab64915.html) antibody (Ser 473) | [Ab81283](http://www.abcam.cn/grk6-antibody-ab64915.html) | Abcam |
| [Anti-ACTB antibody](http://www.abcam.cn/beta-actin-antibody-ab8227.html) | [Ab16039](http://www.abcam.cn/beta-actin-antibody-ab8227.html) | Abcam |
| Anti-Ub antibody | Ab7780 | Abcam |
| [Anti-HA](http://www.abcam.cn/cd97-antibody-c-terminal-ab155798.html) antibody | Ab9110 | Abcam |
| Anti-GAPDH antibody | Ab9485 | Abcam |
